# Psychiatric manifestations of treatable hereditary metabolic disorders in adults

**DOI:** 10.1186/s12991-014-0027-x

**Published:** 2014-09-24

**Authors:** Caroline Demily, Frédéric Sedel

**Affiliations:** 1 Centre for the Detection and Management of Psychiatric Disorders of Genetic Origin, Hospital le Vinatier and UMR 5229 (CNRS and Lyon University), 95 Bld Pinel, Bron 69677, Cedex, France; 2 Federation for Diseases of the Nervous System, Reference Centre for Lysosomal Diseases, Hospital Pitié Salpêtrière, Paris 75013, France

**Keywords:** Inherited metabolic diseases, Psychiatric disorders, Neurological signs, Diagnosis, Treatment, Adults

## Abstract

Detecting psychiatric disorders of secondary origin is a crucial concern for the psychiatrist. But how can this reliably be done among a large number of conditions, most of which have a very low prevalence? Metabolic screening undertaken in a population of subjects with psychosis demonstrated the presence of treatable metabolic disorders in a significant number of cases. The nature of the symptoms that should alert the clinician is also a fundamental issue and is not limited to psychosis. Hereditary metabolic disorders (HMD) are a rare but important cause of psychiatric disorders in adolescents and adults, the signs of which may remain isolated for years before other more specific organic signs appear. HMDs that present purely with psychiatric symptoms are very difficult to diagnose due to low awareness of these rare diseases among psychiatrists. However, it is important to identify HMDs in order to refer patients to specialist centres for appropriate management, disease-specific treatment and possible prevention of irreversible physical and neurological complications. Genetic counselling can also be provided. This review focuses on three HMD categories: acute, treatable HMDs (urea cycle abnormalities, remethylation disorders, acute intermittent porphyria); chronic, treatable HMDs (Wilson’s disease, Niemann-Pick disease type C, homocystinuria due to cystathionine beta-synthase deficiency, cerebrotendinous xanthomatosis); and chronic HMDs that are difficult to treat (lysosomal storage diseases, X-linked adrenoleukodystrophy, creatine deficiency syndrome). We also propose an algorithm for the diagnosis of HMDs in patients with psychiatric symptoms.

## 1 Introduction

Detecting psychiatric disorders of secondary origin is a crucial concern for the psychiatrist. But how can this reliably be done among a large number of conditions, most of which have a very low prevalence? A study performed some time ago [1] involving a sample of 658 patients who had a psychiatric consultation with a complete clinical evaluation and full investigations showed that 9.1% of these patients had a physical condition that presented with psychiatric symptoms. Moreover, such conditions are believed to be the leading cause of psychiatric disorders in the elderly. Metabolic screening undertaken in subjects with psychosis demonstrated the presence of treatable metabolic disorders in a significant number of cases; there were 15 cases out of 268 patients [2]. One in 20 patients might therefore be affected.

The nature of the symptoms that should alert clinicians is also a fundamental issue. A study in adolescents with clinical criteria of catatonia found the presence of a general medical condition in 22% of cases and the presence of a neurodevelopmental disorder in 31% [3].

Several large aetiological groups have been identified among which hereditary metabolic disorders (HMD) are a rare but important cause of psychiatric disorders in adolescents and adults [4]. For example, in 1985, Tishler et al. examined 3,867 psychiatric inpatients in the United States to determine the incidence of acute intermittent porphyria (AIP) and found the rate to be 20 times greater than that of the general population. A retrospective analysis of patients with various HMDs shows that the psychiatric signs may remain isolated for years before other more specific organic signs appear [5]. HMDs that present purely with psychiatric symptoms are very difficult to diagnose because psychiatrists often do not recognise these rare diseases. However, it is important to identify HMDs in order to

 make a definitive diagnosis as early as possible

 refer patients to a referral centre for appropriate management

 initiate specific treatment and prevent the occurrence of irreversible physical and neurological complications—the prognosis of HMDs has been revolutionised by new therapeutic approaches

 provide genetic counselling to the patient and the whole family.

Treatments are available for some HMDs, which we focus on in this article (Table 1). Metabolic decompensation may be prevented and simple; appropriate advice may be provided. In most cases in adults, psychiatric disorders, not limited to psychosis, are the manifestations that point to the presence of HMDs. The diagnosis of the disease ‘at the psychiatric stage’ corresponds to a relatively early phase, and thus, the treatment can have maximum efficacy. If the disease is not picked up at the psychiatric stage, irreversible lesions occur secondarily. It is therefore essential that psychiatrists are aware of these for the differential diagnosis and that opportunities to help the patient are not missed [6],[7].

**Table 1:** Psychiatric manifestations of treatable hereditary metabolic disorders (general overview based on several cases from the literature)

**Psychiatric diagnosis**	**Occurrence**	**Psychiatric symptoms**	**Associated symptoms**	**HMD diagnosis**
Schizophrenia	Triggering factor		Disorders of consciousness, peripheral disease, rapid cognitive decline	Remethylation disorders
	Chronic	Delusions, hallucinations	Juvenile cataract, tendinous xanthomata, cerebellar ataxia, spastic paraplegia, dementia	Cerebrotendinous xanthomatosis
	Chronic, late onset	Hallucinations, behavioural disturbances (+++)	Tetraplegia, spastic paraplegia, cerebellar ataxia, polyneuropathy	Metachromatic leucodystrophia
	Chronic, late onset	Hallucinations, delusions, depression, cognitive decline	Peripheral symptoms, ataxia	GM2 gangliosidosis (Tay-Sachs disease)
Atypical psychosis		Depression, delusions, behavioural disorders	Spastic paraplegia	X-linked adrenoleukodystrophy
	Variable	Confusion, delusions	Ataxia, abnormal movements, supranuclear gaze palsy	Niemann-Pick type C
Inebriation, personality disorder, Guillain-Barre disease, bipolar or schizoaffective disorder	Triggering factor (alcohol, treatment): may be progressive	Behavioural disturbances, impulsivity, depression, mania	Intermittent pain	Acute intermittent porphyria
Bipolar disorder	Chronic	Depression, behavioural disturbances	Extra pyramidal symptoms, dysarthria, akinesia	Wilson’s disease
		Confusion, behavioural disturbances, hallucinations	Headache, abdominal pain, change in diet	Urea cycle disorders
Personality disorder	Chronic	Obsessive compulsive disorder, behavioural disturbances, impulsivity, disinhibition	Ectopia lentis, marfanoid appearance, mental deficiency, thrombosis	Homocysteinuria
	Chronic	Behavioural disturbances, aggressiveness	Mental deficiency, language delay, epilepsy, extrapyramidal symptom	Creatine deficiency syndromes

*HMD* hereditary metabolic disorders.

HMDs can be classified into three categories:

 acute, treatable HMDs

 chronic, treatable HMDs

 chronic HMDs that are difficult to treat.

### 1.1 Acute, treatable HMDs

This group includes urea cycle abnormalities, remethylation disorders and the porphyrias. The acute manifestations often have an element of confusion (sometimes poorly detected and considered as an acute psychotic disorder) and, in certain cases, have a rhythmicity to them.

### 1.2 Urea cycle abnormalities

The urea cycle is the metabolic pathway in the liver that eliminates excess endogenous and exogenous nitrogen through detoxification of ammonia into urea. The diseases of the urea cycle include a group of six distinct enzyme deficiencies of genetic origin, all of which result in dysfunction of the urea cycle and a rise in the serum ammonia level with subsequent abnormalities of blood amino acids (glutamine, ornithine, citrulline and arginine). Ornithine transcarbamylase (OTC) deficiency is the most common as it is an X-linked recessive condition, while the other deficiencies have an autosomal recessive pattern of inheritance.

Hereditary urea cycle deficiencies may present at any age (prevalence 1/10,000) [8]. In addition to forms that present in the neonatal period and in young children, there are forms with a late onset discovered in adolescents or young adults. These diseases may cause confusion, behavioural disorders or hallucinations that may suggest an atypical form of depression [9], an acute psychotic disorder or even resemble schizophrenia [10]. Patients often have protein intolerance and spontaneously change their diet, becoming vegetarians or anorexic [11]. Late-onset metabolic dysfunction may appear to be spontaneous or associated with simple dietary changes involving a higher protein intake (change of milk, meat-based diet). Protein hypercatabolism or the start of a treatment (e.g. with corticosteroids or valproate) is sometimes a trigger. The psychiatric symptoms that occur are nearly always accompanied by headache and/or gastrointestinal symptoms (nausea, vomiting). Treatment based on protein restriction allows acute decompensation to be avoided.

### 1.3 Remethylation disorders

A feature common to remethylation disorders is defective remethylation of homocysteine to methionine (a reaction catalysed by methionine synthetase), usually caused by methylene tetrahydrofolate reductase (MTHFR) deficiency or deficiencies of cobalamin metabolism (CblC) and subsequent functional deficiency of folate or B_12_ despite normal circulating levels.

Remethylation disorders may present at any age and be picked up in adults in three different ways [12]:

 Systematic screening in an asymptomatic patient where a relative is known to be affected

 Detection in the context of a haematological problem (thrombosis in particular)

 Detection following often florid neurological symptoms (psychiatric disorders associated with rapid cognitive decline or even a dementia syndrome, or motor disorders).

The psychiatric involvement may essentially be characterised by psychotic symptoms (behavioural disorders, hallucinations), which may be accompanied by disorders of consciousness and peripheral diseases (subacute paraplegia, peripheral neuropathy and coma). Such episodes may even occur after the age of 50 and may be triggered by a surgical operation. Brain imaging sometimes shows demyelination but may be completely normal. An MRI scan of the spinal cord may show high signal intensity in the dorsal columns of the spinal cord similar to that seen in pernicious anaemia. Early treatment is very effective in all cases and prevents neurological complications.

### 1.4 Acute intermittent porphyria

Traditionally referred to as the ‘royal malady’ as it is popularly believed that the British king George III suffered from it (something that is now disputed: Hift et al. [13]), AIP is an autosomal dominant disease with variable penetrance, linked to deficiency of an enzyme involved in the biosynthesis of haem: porphobilinogen deaminase (PBG deaminase). The prevalence is 10/100,000 in the general population and 21/10,000 in the psychiatric hospital population in the United States [14]. Acute attacks of porphyria are often triggered by porphyrinogenic treatments (oestrogen/progesterone contraceptives, barbiturates, sulfonamides, antiepileptics), sepsis or alcohol ingestion [15]. The most typical manifestations are psychiatric involvement, intermittent pain (especially abdominal pain) and neurological involvement (which may even manifest as tetraplegia and wrongly lead to the diagnosis of Guillain-Barré syndrome).

The attacks usually begin with minor changes in behaviour such as anxiety, impatience or insomnia. Involvement of the peripheral nervous system may occur early on (paralysis, sensory disturbances). There are sometimes noted to be sudden changes in behaviour such as aggressiveness, impulsivity or suicide attempts. Heightened vigilance is required because these symptoms are sometimes under-diagnosed or attributed to a personality disorder (e.g. chronic fatigue, relationship difficulties). Isolated psychotic [16] or catatonic symptoms [15] have also been documented. The monthly occurrence during the luteal phase in women may wrongly point towards a diagnosis of bipolar disorder. Exacerbation by alcohol may resemble the presentation of excessive alcohol intake (acute intoxication). Overall, the diagnosis of acute intermittent porphyria should be considered in any psychiatric syndrome with unexplained pain, especially if the pain is cyclical in nature [17]. Diagnoses are achieved based on urine porphobilinogen measurements; urinary porphobilinogen levels are greatly increased but may be normal between attacks. Symptomatic treatment is based on removal of the exogenous cause (culprit drugs or alcohol) combined with an infusion of haem arginate.

### 1.5 Chronic, treatable HMDs

The category of chronic, treatable HMDs includes Wilson’s disease, Niemann-Pick disease type C (NP-C), some remethylation disorders (homocystinuria due to cystathionine beta-synthase deficiency) and cerebrotendinous xanthomatosis, even though the psychiatric signs are rarely an isolated finding in the latter. In this group of disorders, the psychiatric manifestations usually have a long-term course and have some special features such as the presence of catatonic symptoms or visual hallucinations. In this group, neurological involvement is often more pronounced.

### 1.6 Wilson’s disease

Wilson’s disease is a rare genetic disease (prevalence 1/30,000) with autosomal recessive inheritance [18]. It is the result of mutations of the *ATP7B* gene on chromosome 13, which plays a role in copper metabolism. This disease is characterised by a toxic build-up of copper mainly in the liver and central nervous system. The resulting overload is usually asymptomatic until the age of 6, but inexorably leads to hepatic and neurological degeneration due to the direct toxicity of copper. In most cases, symptoms appear in preadolescent children, adolescents and young adults [19]. Very rare cases have been reported in subjects over the age of 60. A retrospective study in 195 patients found psychiatric symptoms to be present in 51% of cases and could have preceded neurological signs in 20% of cases [20]. The major psychiatric symptoms were behavioural disorders (irritability, aggressiveness, marked changes in personality, disinhibition) or a depressive syndrome, while psychosis was found in only 1% of cases.

Differentiating the disorders from neuroleptic malignant syndrome can sometimes be a tricky process [21]. An association with bipolar disorder has recently been documented [22]. A study using the Neuropsychiatric Inventory (NPI) allowed better characterisation of the psychiatric disorders of patients with Wilson’s disease [23]. These psychiatric disorders may be present from the very start of the disease [24] and may remain isolated for several years [25]. Early detection of the disease and the initiation of chelating therapy or treatment with zinc salts can prevent neurological and hepatic involvement. Treatment at the ‘psychiatric’ stage is therefore essential. The use of antipsychotics should be avoided as far as possible (prescription limited to psychotic disorders or severe behavioural disorders), as they can cause akinesia and rigidity which may progress despite treatment with copper chelators [26].

### 1.7 Niemann-Pick disease type C

NP-C is a lysosomal storage disease associated with an abnormality of cellular lipid transport that results in the accumulation of cholesterol and glycosphingolipids in the brain and other tissues. It is due to mutations in either of the genes, *NPC1* or *NPC2*. The clinical spectrum of NP-C ranges from rapidly fatal visceral forms in neonates to the adult form—a slowly progressive neurodegenerative disease. However, it is very likely that a significant proportion of patients is not diagnosed, or is incorrectly diagnosed, because of the poor awareness of the disease and the relatively non-specific nature of initial clinical signs [27]. Indeed, the large range of neurological or psychiatric signs seen during the course of NP-C can mimic other neurological and psychiatric diseases.

In adult patients, a range of initial psychiatric diagnoses have been recorded, including ‘schizophrenia’, ‘Alzheimer’s disease in a young subject’, ‘frontotemporal dementia’, ‘Parkinson’s disease’, ‘Wilson’s disease’, ‘multiple sclerosis’, ‘Creutzfeldt-Jakob disease’ and ‘Wernicke’s encephalopathy’ [28]. Psychiatric disorders are the most common presentation (38% of patients in the study by Sévin et al. [28]). Psychiatric manifestations may remain isolated for several years. They are usually psychotic in nature (e.g. paranoid delusions, visual or auditory hallucinations, delusions of reference, behavioural disorders with aggressiveness, self-mutilation, social isolation), although depressive syndromes have been reported (e.g. isolated transient visual hallucinations, bipolar disorder or obsessive-compulsive disorder) [29]–[32]. Onset may be gradual or acute, with spontaneous remissions and relapses. Most patients who initially have psychotic symptoms do not have any obvious abnormalities on neurological examination or these abnormalities have been wrongly attributed to the neuroleptic treatment, so much so that most patients are diagnosed with schizophrenia or other forms of psychosis.

Alongside psychiatric disorders, patients develop early motor signs: ataxia, abnormal movements and, an almost consistent finding, vertical supranuclear gaze palsy. This characteristic sign may be difficult to recognise as the condition can be subtle—for a long time, only voluntary saccadic movements are affected, whereas eye tracking remains normal (it may be affected at a later stage). These saccadic eye movements are studied by asking the patient to alternately look to the sides then upwards then downwards on command (without the aid of the examining finger). Slowing of the movements typically starts with the downward motion then affects the upward movements and, eventually, the lateral movements.

The diagnosis of the disease is based on demonstration of the accumulation of free cholesterol in fibroblasts in culture with the help of a specific stain, filipin, and demonstration of specific mutations in the genes *NPC1* and *NPC2*. A case where NP-C was associated with a low ceruloplasmin level has recently been reported, making it difficult to distinguish from Wilson’s disease [33].

Treatment with miglustat (Zavesca®; Actelion Pharmaceuticals Ltd.) reduces neuronal sphingolipid accumulation and slows or delays neurological disease progression [34],[35]. Other treatments are symptomatic in nature. It is important that the disease is detected as early as possible, especially in the more atypical adult forms [36].

### 1.8 Homocystinuria

Homocystinuria is a very rare autosomal recessive hereditary disease (prevalence 1/200,000) of methionine metabolism due to the deficiency of cystathionine beta-synthase, which is encoded by the gene *CBS* (21q22.3) [37]. Causal mutations in this gene lead to abnormal methionine metabolism, with subsequent accumulation of homocysteine in the blood and cysteine deficiency. It is characterised by very high plasma total homocysteine levels (over 100 μM/L).

The vascular system, eyes and nervous system are affected in the vast majority of cases. The most typical symptoms are skeletal abnormalities with a Marfanoid appearance, intellectual impairment, ectopia lentis and an increased risk of thrombosis and haematological disorders. Psychiatric complications are found in 51% of adult patients [38] and include behavioural disorders (e.g. physical violence, drug or alcohol abuse), personality disorders (hyperactivity, excessive spending and disinhibition), depression and obsessive-compulsive disorder. However, cases of psychosis or schizophrenia have been shown to be uncommon in large cohort studies [38]–[40].

Laboratory investigations that need to be done if the condition is suspected include assays of plasma total homocysteine (increase), plasma methionine (increase) and urinary homocystine (low). An ophthalmology examination to look for ectopia lentis is also very informative.

### 1.9 Cerebrotendinous xanthomatosis

Cerebrotendinous xanthomatosis is a hereditary metabolic disorder secondary to mutations of the gene *CYP27A1* (prevalence 1–9/100,000), situated on the long arm of chromosome 2; approximately 50 mutations have been identified to date [41]. Cerebrotendinous xanthomatosis is characterised biochemically by sterol 27-hydroxylase deficiency. This enzyme is involved in the degradation of cholesterol. The metabolic deficiency causes gradual build-up of cholesterol and especially its metabolite, cholestanol, in various tissues including the brain and tendons. Clinically, patients usually have juvenile cataract and tendinous xanthomata associated with neurological signs (cerebellar ataxia, spastic paraplegia and dementia) and psychiatric disorders (psychotic manifestations, hallucinations) [42]. Diagnoses are usually achieved during adulthood, which represents missed opportunities for early treatment in many cases. Laboratory investigations that should be conducted if cerebrotendinous xanthomatosis is suspected include assay of cholestanol and, in particular, determination of the cholesterol/cholestanol ratio (normal <1:1,000). Treatment with chenodeoxycholic acid allows stabilisation of, or improvement in, the psychiatric signs.

Bonnot et al. [43] reported a case of a sibship for which an atypical psychiatric presentation associated with polyneuropathy and pyramidal signs led to the diagnosis of cerebrotendinous xanthomatosis. The initiation of treatment with chenodeoxycholic acid resulted in marked improvement in behaviour, leading to re-socialisation.

### 1.10 Chronic HMDs that are difficult to treat

#### 1.10.1 Lysosomal storage diseases

Several lysosomal storage diseases may manifest themselves with psychiatric disorders in adults. These purely psychiatric presentations are seen in metachromatic leukodystrophy and late-onset GM2 gangliosidosis.

Metachromatic leukodystrophy is due to deficiency of arylsulfatase A, an enzyme encoded by the gene *ARSA*, giving rise to accumulation of urinary sulfatides (prevalence 1/50,000 to 1/170,000, autosomal recessive mode of inheritance) [44]. It is characterised by periventricular leukodystrophy leading to tetraparesis. The disease most often presents in childhood (in 60% of cases) but can occur in adults (20% of cases) and sometimes even late in life (aged 60+ years).

In terms of genotype, late-onset forms are thought to have different molecular bases [45]. In this case, the psychiatric symptoms are often present from the outset and may mimic schizophrenia with hallucinations and behavioural disorders [46]. The clinical presentation deteriorates after several years, with neurological involvement (dementia, spastic paraparesis, cerebellar ataxia, convulsive seizures), optic nerve atrophy and a characteristic but often asymptomatic demyelinating polyneuropathy. In monosymptomatic forms, isolated polyneuropathy or initial psychiatric disorders are present.

Two genetically distinct adult forms with different initial presentations have been described: 1) an initially motoric form associated with homozygous *P426L* mutations and characterised by cerebellar ataxia, paraparesis and then progressive cognitive decline with psychiatric disorders; and 2) a form with a psychiatric onset in patients who are heterozygous carriers of I179S mutations, during which behavioural disorders that may mimic schizophrenia are complemented by a dementia syndrome and motor disorders (paraparesis, cerebellar syndrome) [47].

Brain MRI studies demonstrate diffuse periventricular leukoencephalopathy. The aetiological diagnosis is based on assay of arylsulfatase A in leucocytes. However, this diagnostic investigation is not sufficient alone, as 1%–2% of the population has pseudodeficiency of arylsulfatase A. There is currently no specific treatment for this disease, but several therapeutic trials involving the use of enzyme replacement therapy are in progress.

GM2 gangliosidosis is a sphingolipidosis associated with hexosaminidase deficiency. GM2 gangliosidosis may present in adulthood. Late-onset forms are sometimes called late-onset Tay-Sachs disease, despite the fact that minimal signs can initially appear during childhood. GM2 gangliosidosis presents with neurological disorders (peripheral motor involvement, cerebellar ataxia or dystonia) associated with psychiatric signs in 20%–40% of cases (e.g. acute psychotic disorder, hallucinations, depressive syndrome) [48]–[50]. These signs may remain isolated for years before the appearance of neurological signs but can appear later. The occurrence of psychiatric disorders associated with rapid cognitive decline, with executive and verbal deficits dominating, should alert the physician [51]. The neurological signs are often preceded by precursor symptoms in childhood: balance disorders, awkwardness, difficulty in climbing stairs and recurrent psychiatric disorders (psychotic or bipolar) [50]. Dystonia, supranuclear gaze palsy, painful sensory polyneuropathy and neuroautonomic disorders (hyperhidrosis) sometimes complicate the syndrome, making the diagnosis more difficult. Brain MRI studies may be normal or demonstrate cerebellar and cortical atrophy. Diagnoses are usually based on measurement of the activity of hexosaminidases A and B in leucocytes or fibroblasts. Tricyclic antidepressants and phenothiazines have little efficacy and may worsen the psychiatric signs. Miglustat has also been used in this indication, so far without convincing results [52].

### 1.11 X-linked adrenoleukodystrophy

X-linked adrenoleukodystrophy (X-ALD) is a rare genetic disease affecting peroxisomal metabolism of very long chain fatty acids (VLCFAs) due to mutations in the gene *ABCD1* located on Xq28. The incidence of X-ALD is approximately 1/17,000 births, making it the commonest of the peroxisomal disorders [53].

The classic cerebral form of X-ALD presents in childhood with progressive demyelination of the peripheral and central nervous system, adrenal insufficiency and accumulation of VLCFAs in the plasma and tissues; 30%–40% of affected males and over half of carrier females may develop adrenomyeloneuropathy in adulthood, characterised by progressive spastic paraplegia [54]. The majority of affected adult patients described to date had psychiatric signs that preceded motor signs by many years [55]. The psychiatric signs present as gradual changes in behaviour, mania, depression and acute psychotic episodes [56],[57].

### 1.12 Creatine deficiency syndromes

Creatine deficiency syndromes are characterised by intracerebral creatine deficiency due to deficiencies of creatine synthesis (deficiency of AGT or GAMT) and of the creatine transporter (SLC6A8 deficiency). The transporter deficiency makes the disease very difficult to treat. These diseases feature severe intellectual impairment, with severe language delay, which may be variously accompanied by epilepsy, an extrapyramidal syndrome and behavioural disorders. Most of the time, they are detected in children, but adult forms have been documented. Carriers of mutations of the creatine transporter gene (*SLCA8*) may have mild intellectual impairment and behavioural disorders (mainly aggressiveness) [58]. Their diagnosis is possible by measurement of creatine and guanidinoacetate in biological fluids.

### 1.13 How can screening be done?

**Figure 1:**
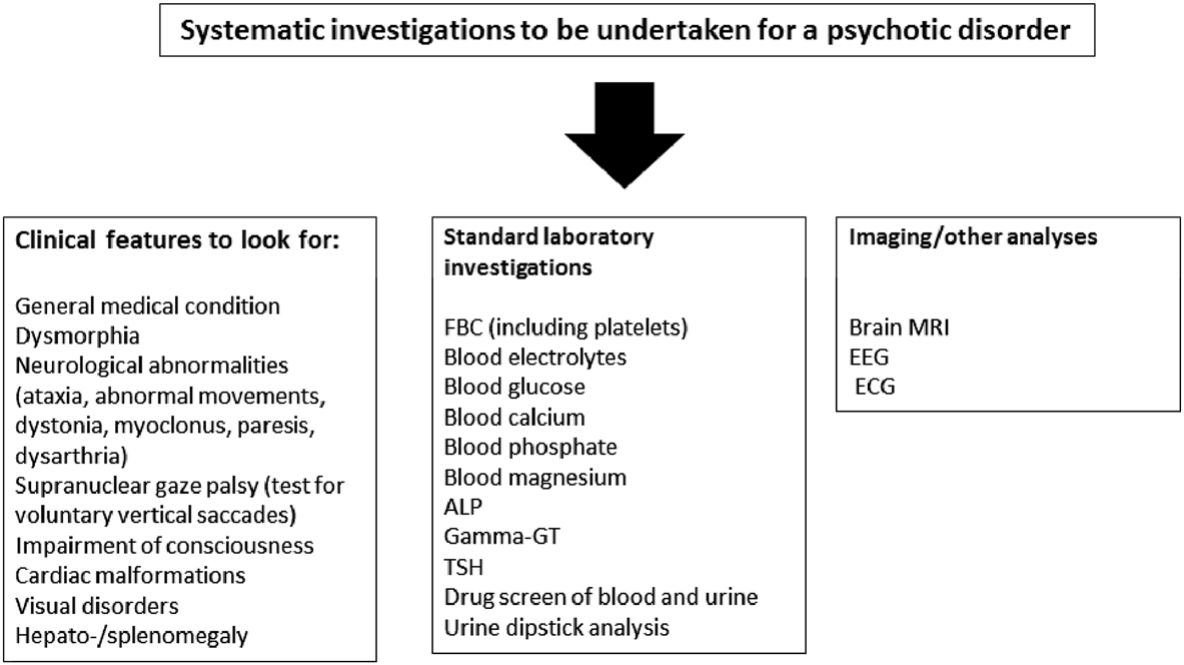
**Systematic screening in psychotic disorders.***ALP* alkaline phosphatase, *TSH* thyroid stimulating hormone, *MRI* magnetic resonance imaging, *EEG* electroencephalography, *ECG* echocardiography.

Screening for HMDs in a population of individuals with mental disorders is most complex when the disorder occurs in an isolated manner in a patient with no personal or family history of a metabolic disorder. Moreover, the presence of minor neurological signs in patients with mental disorders can complicate the detection of something that may be of metabolic origin. Yet, it is at this stage that the diagnosis and resulting management can completely change the patient’s course and a systematic screening can be proposed (Figure 1).

Should systematic screening be offered? This does not appear feasible, particularly as the laboratory investigations traditionally suggested for screening for HMDs may be ineffective (neither sensitive nor specific) in a population of individuals with psychiatric disorders (personal communication). This finding is easily explained by the influence of numerous confounding factors: psychotropic treatments, extremely sedentary lifestyle, disordered diet, use of toxic substances, etc. Resistance to treatment and even worsening of the disorders should also serve as an alarm signal. If atypical symptoms are present, a simple diagnostic algorithm should comprise the following: first-rank symptoms (visual hallucinations, mental confusion, catatonia, fluctuation of symptoms, unusual (or paradoxical) response to treatment, progressive cognitive change) associated with second-rank symptoms (early onset, acute onset, intellectual impairment, lack of treatment efficacy) which may aid the psychiatrist in detecting an HMD [59].

**Figure 2:**
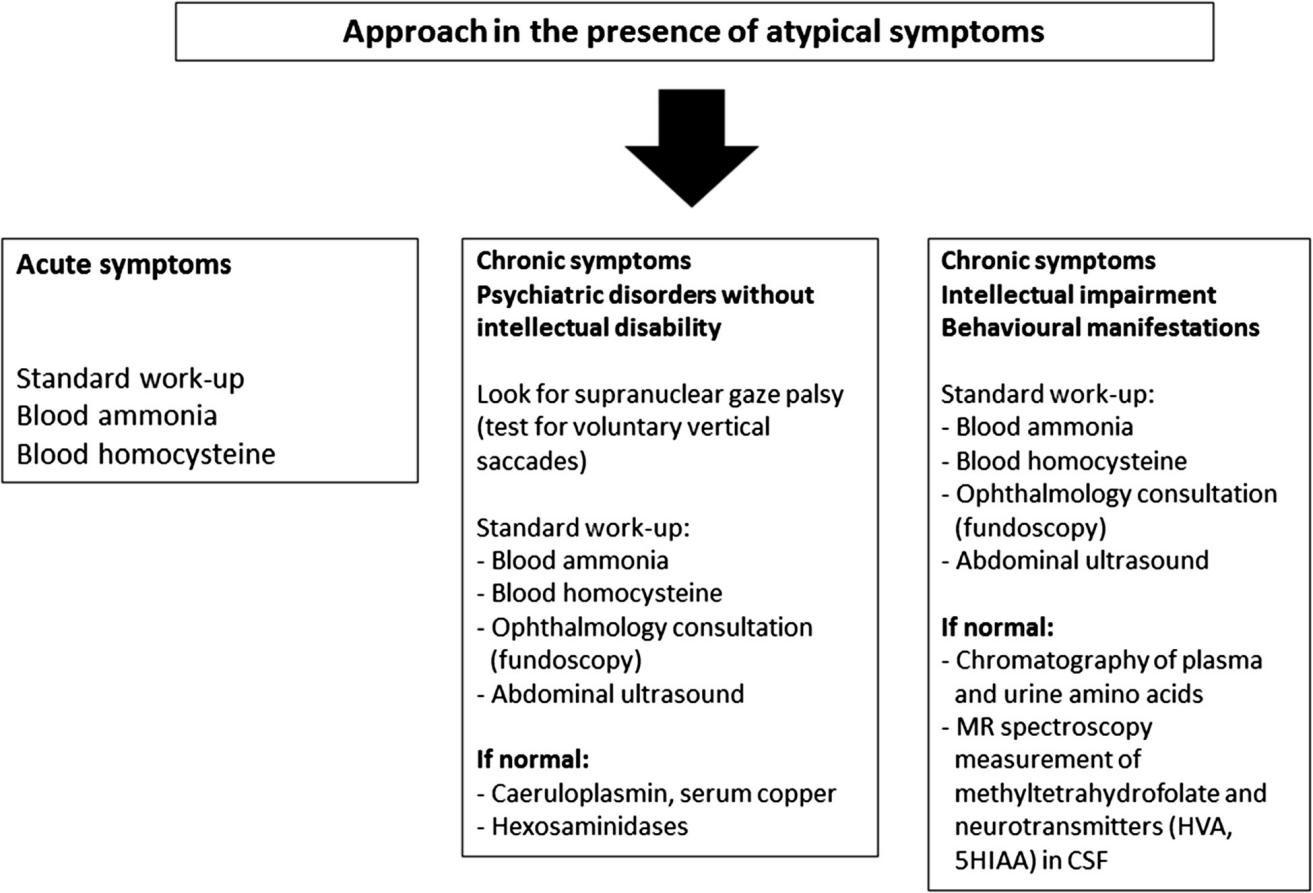
**Algorithm for the diagnosis of hereditary metabolic disorders in the presence of atypical symptoms.***CSF* cerebrospinal fluid, *HVA* homovanillic acid, *MR* magnetic resonance, *SHIAA* 5-hydroxyindoleacetic acid.

Screening for these diseases is therefore very specific and requires specialist advice (Figure 2). However, having said that, it is important to alert psychiatrists and general practitioners to think about these conditions. In current practice, the management of psychotic disorders must always be accompanied by a specific work-up.

## 2 Conclusion

Unfortunately, not all patients with an HMD benefit from a diagnosis or specific treatment in the psychiatric setting despite the fact that there are referral centres specialising in these conditions. This shortcoming may partly be explained by the complexity of these diagnoses and the fact that they are rare. The psychiatric signs that suggest these disorders are usually non-specific, and it is the general physical context that points towards the correct diagnosis.

Considerable work remains to be done to describe these disorders since knowledge about the presentation of these diseases in adults has not yet been clearly documented. Every day, new case reports are described that all have special features and variable modes of presentation.

The metabolic approach to diagnosing mental disorders is a fast-expanding area and, in years to come, will probably alter the diagnostic process in psychiatry. However, the very history of psychiatry, where for many years those involved in treatment called for a non-medical model, making the traditional distinction between treatments for the mind and physical medicine, also partly explains the lack of knowledge in this field. It is now essential that geneticists and neurologists on the one hand and psychiatrists on the other are able to collaborate in the management of adult patients.

## Abbreviations

ALP: alkaline phosphatase

CBS: cystathionine beta-synthase

ECG: electrocardiography

EEG: electroencephalography

FBC: full blood count

HMD: hereditary metabolic disorders

MRI: magnetic resonance imaging

MTHFR: methylene tetrahydrofolate reductase

NP-C: Niemann-Pick disease type C

OTC: ornithine transcarbamoylase

PBG: porphobilinogen

TSH: thyroid stimulating hormone

VLCFAs: very long chain fatty acids

X-ALD: X-linked adrenoleukodystrophy

## Competing interests

FS has received consultancy fees and research funding from Actelion Pharmaceuticals Ltd.

## Authors’ contributions

CD and FS wrote the first draft of this manuscript, had significant input into each subsequent draft, and approved the final draft for submission. Both CD and FS are accountable for all aspects related to the accuracy or integrity of this work. Both authors read and approved the final manuscript.
